# Paratesticular superficial angiomyxomas: A case report

**DOI:** 10.1097/MD.0000000000043521

**Published:** 2025-08-01

**Authors:** Yi Huang, Tang Tielong

**Affiliations:** a Department of Urology, People’s Hospital of Pengshan District, Meishan, Sichuan, China; b Department of Urology, Affiliated Hospital of North Sichuan Medical College, Nanchong, China.

**Keywords:** case report, paratesticular, superficial angiomyxomas

## Abstract

**Rationale:**

Superficial angiomyxoma (SA) is a rare benign tumor that occurs in the superficial dermis or subcutaneous tissue. It is characterized by a significant presence of mucous matrices and blood vessels. In case reports, nodules typically appear on the trunk, head, and limbs. We conducted a search of PubMed, Embase, the Cochrane Library, and other medical databases, finding only a limited number of case reports. Here, we present the case of a 43-year-old male with paratesticular SA, and share our therapeutic experience.

**Patient concerns:**

A 43-year-old man was admitted to the hospital due to an enlarged scrotum.

**Diagnoses:**

We surgically excised the mass. Histopathological examination using hematoxylin and eosin staining revealed abundant myxoid stroma. Immunohistochemical staining was performed to establish a differential diagnosis of deep (aggressive) angiomyxoma. Staining for CD34, desmin, estrogen receptor, and vimentin was positive, while S-100 was negative. Based on these findings, the patient was diagnosed with SA.

**Interventions:**

In our case, the tumor boundary was well-defined, and we successfully removed it in its entirety.

**Outcomes:**

We have been monitoring the patient for 3 years, and there has been no recurrence of the condition.

**Lessons:**

To prevent the recurrence of SA, complete resection is recommended, including partial resection of normal tissue.

## 1. Introduction

Superficial angiomyxoma (SA) is a rare, benign tumor that occurs in the superficial dermis or subcutaneous tissue. They consist of a large number of mucous matrices and blood vessels. In case reports, nodules generally appeared in the trunk, head, and limbs.^[1]^ SA was first described by Carney et al. It is associated with an autosomal dominant disease called the Carney syndrome.^[2]^ A rare autosomal dominant genetic disorder is characterized by multiple myxomas present in the skin, mammary glands, and heart with endocrine overactivity.^[3]^ SA generally only presents with obvious mass symptoms.^[4]^ Therefore, they should be differentiated from perineal cysts, lipomas, leiomyomas, leiomyosarcomas, liposarcomas, testicular tumors, and other unidentified masses. Ultrasound doctors usually predict common benign tumors, and the surgeon simply removed them for examination.

## 2. Case presentation

**Figure 1.**
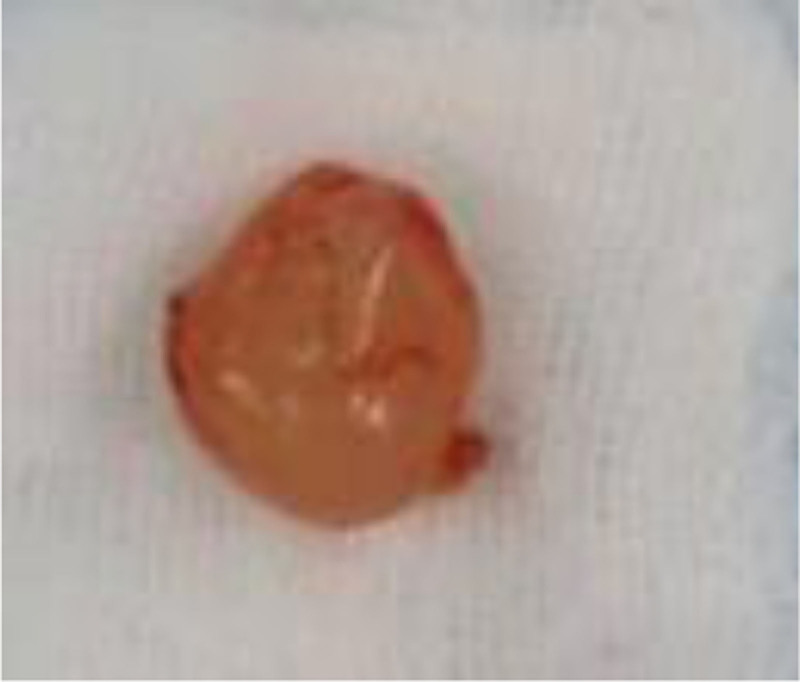
Specimen photograph. The resected specimen measured approximately 2.5 cm × 1.5 cm × 1.3 cm, with well-defined margin.

**Figure 2.**
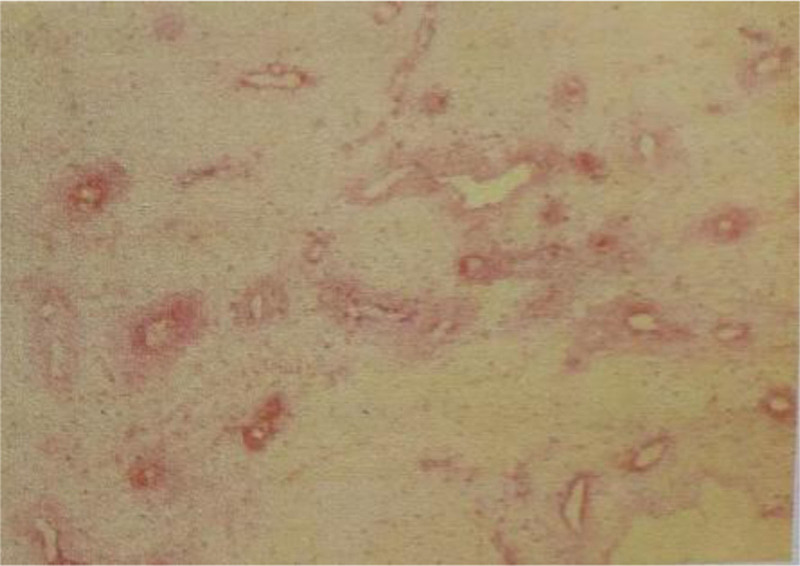
Photomicrograph of the specimen. The specimen was a well-bounded mass with uniform, mucus rich soft tissue tumor cells (H&E, ×100). H&E = Hematoxylin and Eosin.

**Figure 3.**
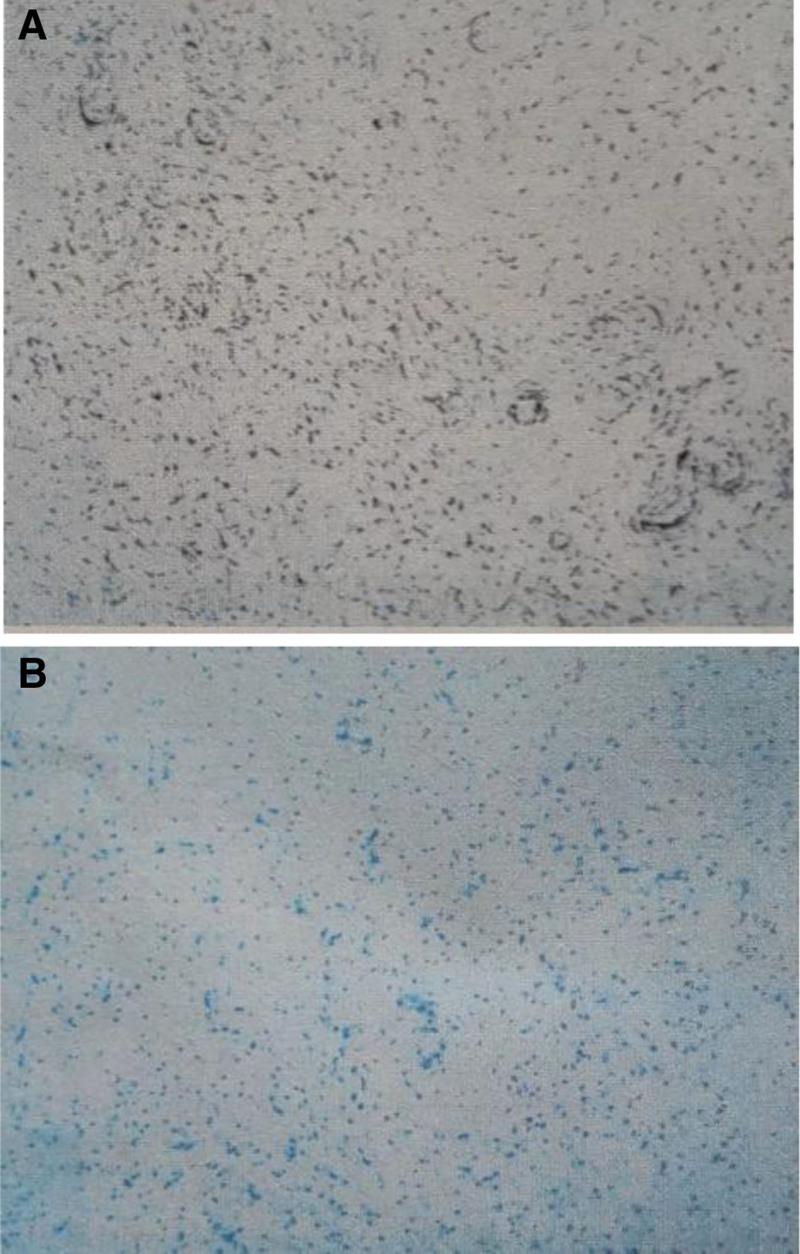
Photomicrograph of the specimen. Immunohistochemical stain was positive for desnin (A, ×100) and ER (B, ×100). ER = estrogen receptor.

A 43-year-old man confessed that there was no obvious induction of a mass in the right scrotum 3 months ago. It was approximately 3 cm in size, oval shaped, and soft, and had no history of special skin or family history. Scrotal ultrasonography revealed a solid hypoechoic mass.Does not rule out the possibility of paratesticular adenoma? We surgically excised the mass. The completely excised mass was 2.5 cm × 1.5 cm × 1.3 cm in size and had a bright yellow color and translucent surface (Fig. 1). Histopathological examination with hematoxylin and eosin staining revealed abundant myxoid stroma (Fig. 2). Immunohistochemical staining was performed to establish a differential diagnosis of deep (aggressive) angiomyxoma. Staining with CD-34, desnin, estrogen receptor, and vimentin was positive, and S-100 was negative (Fig. 3). On the basis of these findings, the patient was diagnosed with SA. So far, we have been followed up for 3 years, and the patient has not experienced recurrence.

## 3. Discussion

SA is a rare, benign tumor that occurs in the superficial dermis or subcutaneous tissue. They consist of a large number of mucous matrices and blood vessels. SA was first described by Carney et al. It is associated with an autosomal dominant disease called the Carney syndrome.^[1]^ A rare autosomal dominant genetic disorder is characterized by the presence of multiple myxomas in the skin, mammary glands, and heart.^[2]^ Endocrine overactivity, such as Cushing’s syndrome and acromegaly, can also be associated with SA due to pigmented adrenocortical tumors and pituitary adenomas.^[3]^

Allen et al^[2]^ reported cases of SA occurring without the Carney complex and based on the clinicopathological and immunohistochemical features of SA, Calonje et al^[4]^ reported that SA and the Carney complex are independent of each other. Paratesticular SA is extremely rare. In case reports, nodules generally appeared in the trunk, head, and limbs.^[5]^ This patient did not demonstrate other manifestations of Carney syndrome or family history. Through postoperative pathologic and immunohistochemical examinations, we confirmed that the mass was an SA.

SA generally only presents with prominent mass symptoms.^[6]^ Therefore, they should be differentiated from deep aggressive angiomyxoma, perineal cysts, lipomas, leiomyomas, leiomyosarcomas, liposarcomas, testicular tumors, and other unidentified masses. Deep aggressive angiomyxomas are found in the subcutis, deep-seated and exhibit an infiltrative, diffuse growth pattern. The diagnosis of SA is histological because it lacks distinctive clinical manifestations.^[7]^ Histopathological examination with hematoxylin and eosin staining reveals abundant myxoid stroma. Immunohistochemistry shows CD-34, desnin, estrogen receptor, and vimentin is positive, and S-100 was negative. It is of great significance for clinical diagnosis, treatment and prognosis evaluation to clarify the differences between paratesticular SA and other masses.

Ultrasound doctors usually predict common benign tumors, and the surgeon simply removed them for further examination. SA has no metastatic potential and exhibits a low recurrence rate following complete excision.^[4]^ The primary causes of local recurrence include incomplete resection and deep invasion of the SA.^[8]^ To prevent recurrence of SA complete resection is recommended, including partial removal of surrounding normal tissue.^[9]^ However, Chan et al reported that recurrence is not correlated with positive margins.^[10]^ In our case, the tumor boundary was clear and we removed it completely. The current plan for follow-up and surveillance is for routine, annual physical examinations, given its benign nature and the fact that his initial lesion was easily found on physical examination. We have been followed up for 3 years and the patient has not experienced recurrence.

## Author contributions

**Writing** – **original draft:** Yi Huang.

**Writing** – **review & editing:** Yi Huang, Tang Tielong.
